# Histone Deacetylase Inhibitory and Cytotoxic Activities of the Constituents from the Roots of *Sophora Pachycarpa*

**DOI:** 10.22037/ijpr.2020.112442.13760

**Published:** 2020

**Authors:** Saba Soltani, Motahareh Boozari, Samad Nejad Ebrahimi, Gholam Reza Amin, Mehrdad Iranshahi

**Affiliations:** a *Department of Pharmacognosy, Faculty of Pharmacy, Tehran University of Medical Sciences, Tehran, Iran. *; b *Department of Pharmacognosy, School of Pharmacy, Mashhad University of Medical Sciences, Mashhad, Iran. *; c *Department of Phytochemistry, Medicinal Plants and Drugs Research Institute, Shahid Beheshti University, Evin, Tehran, Iran. *; d *Biotechnology Research Center, Pharmaceutical Technology Institute, Mashhad University of Medical Sciences, Mashhad, Iran.*

**Keywords:** Sophora pachycarpa, Histone deacetylase inhibitors, prenylated flavonoids, Cytotoxicity, Molecular docking

## Abstract

Four prenylated flavonoids, including isosophoranone, sophoraflavanone G, alopecurone J, alopecurone P, and a resveratrol derivative HPD (2-(4-hydroxyphenyl)-2,3-dihydrobenzo[b] furan-3,4,6-triol), were isolated from the roots of *Sophora pachycarpa*. The cytotoxic activity of obtained compounds was evaluated against A2780, A549, HeLa, and HCT116 human cancer cell lines. We also evaluated their histone deacetylase (HDAC) inhibitory activities. Of all compounds tested, alopecurone J was the most active with IC_50_ values in the range of 9.97−30.91 μM against four cancer cell lines with potent pan-HDAC inhibitory activity (IC_50 _= 0.08−3.85 μM). Molecular docking experiments of these compounds with HDAC8 displayed potential selective HDAC inhibitory. Molecular docking data showed consistent results in the *in-vitro *experiments with high selectivity towards HDAC8. The Resveratrol group plays an essential role in HDAC inhibition.

## Introduction

HDACs are enzymes that cleave acetyl groups from acetyl-lysine residues in histones and various nonhistone proteins. Both acetylation and deacetylation of histones play a fundamental role in epigenetic regulation of gene expression, and disruption of the balance between HATs and HDACs causes many disorders and metabolic diseases (1, 2). Recent evidence shows that cancer is associated with identifiable epigenetic changes, so epigenetic therapy is hypothesized to be a viable gene therapy strategy (3). Many small molecules as HDAC inhibitors are now undergoing clinical trials, and it is urgent to identify agents with high efficacy and low toxicity for application in overexpression of HDACs that generate tumorigenesis. To date, five HDAC inhibitors including vorinostat (Zolinza; Merck & Co.), belinostat (Beleodaq; Spectrum Pharmaceuticals), depsipeptides [romidepsin (Istodax; Celgene), chidamide (Epidaza), and panobinostat (Farydak, Novartis)] have been approved for the treatment of cutaneous cancers or peripheral T cell lymphoma along with nonapproved hydroxamic acids (trichostatine. A), benzamides (entinostat, mocetinostat, tacedinaline) and short chain fatty acids (valproic acid) (4). They derive from both natural sources and synthetic routes. However, primary and secondary resistance to these drugs is common, and it is urgent to identify agents with high efficacy and low toxicity (5). There are intense interests in discovering specific inhibitors from an endless natural source of novel chemotypes and pharmacophores against HDACs.

The genus *Sophora* (Papilionaceae) is an abundant source of prenyl flavonoids (6). The roots contained 9–12% (and the root bark 22–25%) phenolic pigments, flavonoids, steroid glucosides, etc. (7, 8). In 2014, Mousavi and coworkers showed that *S. pachycarpa *extract has a cytotoxic effect on various tumor cell lines via apoptosis induction (9). Based on these findings, *S. pachycarpa* would be a good candidate to search for bioactive components and their application as anti-cancer potential. In our previous study, three known compounds (sophoraflavanone G, alopecurone J and a resveratrol derivative HPD), with one new compound (alopecurone P) were isolated from *S. pachycarpa* root and the mechanism of cytotoxic effect against MCF-7 cell line was investigated (10). In this article, we isolated five compounds and evaluated the cytotoxic effect of these compounds against four different cancer cell lines including human cervical (HeLa), colon (HCT116), ovarian (A2780), and lung (A549) and their inhibitory activities of pan HDAC with the combination of *in silico* methods for evaluating HDAC inhibitory effect.

## Experimental

*General experimental procedures*

Nuclear magnetic resonance (NMR) spectra were obtained using Bruker AVANCE-300 spectrometer (Bruker, Germany). Chemical shifts are given as δ (ppm) using TMS as the internal standard. Semipreparative HPLC was performed on a KNAUER liquid chromatography system with a quaternary pump (Smartline Pump 1000) and a semi-prep c18 column (onyx monolithic; 100×10 mm). Diode array detector (Smartline DAD 2800) and EZ Chrom Elite software were used for the detection and processing of data, respectively. Observation of the plates was carried out under UV CAMAG spectrometer (CAMAG instruments, Berlin, Germany) at 254 nm. Column chromatography was conducted with Si gel 230–400 mesh (Merck, Germany). Analytical TLC was performed on Silica gel 60 F_254_ (Merck, Germany) and preparative TLC was performed on Silica gel 60 GF_254_ (Merck, Germany). 

*Plant material*

The roots of *S. pachycarpa* were collected and identified in April 2015, from Khorasan-Razavi province, the road of karde. A voucher specimen (No. 13247) was deposited in the herbarium of the School of Pharmacy, Mashhad University of Medical Sciences.

*Extraction and isolation*

The total plant extract was obtained from the dried and milled roots of *Sophora pachycarpa *(500 g) with MeOH (for three days per extraction) using the maceration method at ambient temperature. The combined extracts were concentrated to dryness under vacuum pressure to afford 50 g of a brown residue. A part of the extract (35 g) was subjected to silica gel chromatography (230–400 mesh, 55 × 5 cm) using petroleum ether–ethyl acetate and ethyl acetate–methanol [(1:0 to 0:1 and 1:1, 0:1, v/v) × 2L] as a gradient solvent system to give ten fractions (Fr. 1–10). Fraction 4 was further purified by PTLC to give 1 (5 mg, Rf 0.33) using chloroform/methanol (3:0.5) system to yield a yellow powder. Other compounds (2-5) were isolated as previously described (10) using Sephadex LH-20 (25-100 μm, Fluka) CC (MeOH) and high-performance semi-preparative liquid chromatography with a gradient of MeOH–H_2_O (20:80, v/v) on an Onyx monolithic semi-prep C18 (100 × 10 mm) column. The structures of the compounds were confirmed by comparison of ^1^H-and ^13^C-NMR spectra with those of previous reports (10-14).

*Cancer cell lines*

 Human cervical (HeLa), colon (HCT116), ovarian (A2780), and lung (A549) cancer cells, obtained from Biotechnology laboratory stocks, Biotechnology research center (Mashhad, Iran) and National Cell Bank of Iran (NCBI), Pasteur Institute of Iran, Tehran (Iran). 

*Cytotoxic assay*

Compounds 1-5 were tested against HeLa, HCT116, A2780and A549 human cancer cell lines using a nonfluorescent substrate, AlamarBlue® (BioSource Invitrogen, Paisely, UK) and subjected to cell viability assay as described with some modification (15). The cell lines were cultured in RPMI-1640 medium (Cambrex Bioscience) supplemented with 10% fetal bovine serum (FBS), and 1% penicillin/streptomycin at 37 °C in an atmosphere of 95% O_2_ and 5% CO_2_. The cells were seeded in 96-well microplates (1 × 10^4^ cells/100 µL in each well) and cultivated for 24 h before the addition of test compounds. Three wells for each concentration were seeded and triplicate plates were used. The test compounds were dissolved in dimethyl sulfoxide (the final amount of DMSO per well was maintained at 0.1%V/V) and their concentrations adjusted to 100, 50, 25, 12.5, 6.25 μM through dilution with the growth medium. Then, the cells were incubated with the different concentrations of the test compounds, positive control and cell culture medium alone as a negative control for 48 h. After that, 10 μL of alamarBlue® reagent was added to the attached cells and incubated for another four hour. The absorbance was measured at 600 nm using an ELISA microplate reader (Epoch; Bio Tek, Winooski, VT, USA), and the percent viability of the cells was investigated relative to the negative control that was exposed to the culture medium without compounds of the plant. Then, IC_50_ values were calculated by nonlinear regression analysis using GraphPad Prism (Version 6.0) software. The positive control was vorinostat (purity ≥ 98%; Sigma-Aldrich, USA).

*Whole-cell HDAC inhibition assay*

The cellular HDAC assay was based on an assay published by Marek *et al*. (16). HCT116 and HeLa human cancer cell lines were seeded in 96-well tissue culture microplates (1.5 × 10^4^ cells/90 µL culture medium in each well). After 24 h, the cells were incubated for 18 h with increasing concentrations of test compounds, vorinostat as positive control and cell culture medium alone as a negative control. The test compounds were dissolved in dimethyl sulfoxide (the final amount of DMSO/well was maintained at 0.1% V/V) and their concentrations adjusted to 70, 40, 20, 5, 1 μM through dilution with the growth medium. The reaction was started by adding 10 µL of 3 mM Boc-Lys (ε-Ac)-AMC (Bachem, Switzerland) to each well at a final concentration of 0.3 mM. The cells were incubated with the Boc-Lys (ε-Ac)-AMC for 3 h under cell culture conditions. After this incubation, 100 µL/well of stop solution (25 mM Tris-HCl (pH 8), 137 mM NaCl, 2.7 mM KCl, 1 mM MgCl_2_, 1% NP40, 2.0 mg/mL trypsin, 10 µM vorinostat) was added and the mixture was treated for three hour under cell culture conditions. The final volume of the assay is 210 µL in all the wells. After that, the solution was transferred into a black 96-well microplate with flat clear bottom and fluorescence intensity was detected at an excitation of 360 nm and emission of 470 nm in a NOVO star microplate reader (BMGLabTech, Offenburg, Germany).

*Trypsin inhibitory assay*

Trypsin inhibition assay was carried out according to the method of Zwick *et al*. (17). Reactions were carried out in 96-well microplates in triplicate. 2.0 mg/mL trypsin was dissolved in assay buffer (25 mM Tris at pH 8.0 adjusted with HCl, 137 mM NaCl, 2.7 mM KCl, 1 mM MgCl_2_) with vorinostat (10 μM) and added to each microplate well, except for blanks. Test compounds were diluted in DMSO and added at a final concentration of 70 μM (1% DMSO) in each well. The reaction was initiated by the addition of BOC-Lys-AMC substrate (Bachem, Switzerland). The plate was then incubated at 25 °C for 45 min. Trypsin inhibition was calculated by comparing the amount of deacetylated substrate between control and test samples. The relative amounts of deacetylated substrate were obtained by fluorescence reading with excitation at 360 nm and emission at 470 nm. Caffeic acid was used as a positive control.

*Molecular docking studies*

The crystal structures of HDAC8 [PDB entry code: 1T64] was obtained from the Protein Data Bank (PDB). The co-crystallized ligands, all water and non-interacting ions were removed from the receptor. Then all missing hydrogens and sidechain atoms were added. In the next step, Gasteiger charges were calculated for the system. In the final step, by using OPLS3, the receptor was minimized and optimized. The active site of HDAC8 was defined as 10.0 Å radius circles around co-crystal ligand (trichostatin A). Other docking parameters utilized in the program were kept default. For ligand setup, these ligands were optimized with the OPLS3 (Optimized Potential for Liquid Simulations) level. Molecular docking studies were performed with XP module of Glide (Schrödinger LLC, New York). Visualizing was performed by chimera (18). The prediction of drug like properties was carried out using QikProp and pharmaceutical parameters about oral bioavailability were reported. 

## Results

**Figure 1:**
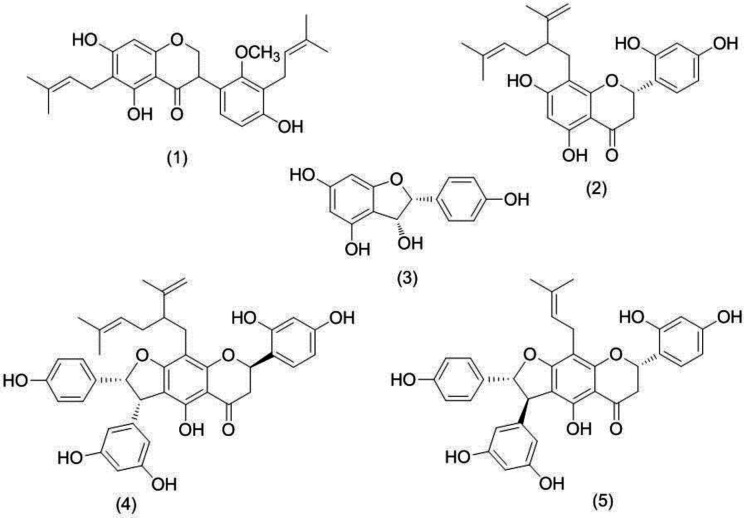
Structures of isosophoranone (1), sophoraflavanone G (2), 2-(4-hydroxyphenyl)-2,3-dihydrobenzo[b]furan-3,4,6-triol (3), alopecurone J (4) and alopecurone P (5).

The MeOH extract of the roots of *S. pachycarpa* was subjected to silica gel column chromatography. Earlier fractions (Fr.4, 6) were further purified by the preparative thin-layer chromatography (PTLC) to obtain isosophoranone (**1**) and a lavandulyl flavanone, sophoraflavanone G (**2**) (13, 19). Purification of Fr. 8 through Sephadex LH-20 gave a resveratrol derivative HPD (2-(4-hydroxyphenyl)-2, 3-dihydrobenzo [b] furan-3, 4, 6-triol) (**3**); and further purification by semipreparative RP-HPLC gave two flavanone stilbenes, alopecurone J and P (**4**, **5**) (10, 11, 14). The structures of 1-5 were identified by comparing their spectroscopic data with the literature (Figure 1). 

As in our previous study (20), the antiproliferative activity of the compounds (**1-5**) was evaluated against human cancer cell lines (HeLa, HCT116, A2780 and A549) by AlamarBlue® assay (Table 1). 

The 50% inhibitory concentration (IC_50_) values were in the range of 9.97-76.54 μM against all cell lines. The data suggested that test compounds had selective cytotoxic activity against the cancer cell lines. Of all compounds tested, alopecurone J (4) was the most active with IC_50_ values in the range of 9.97−30.91 μM against four cancer cell lines. Since the isolated compounds showed more cytotoxic effects in human colon cancer HCT116 and human cervical HeLa cell lines in comparison to other cell lines, *in-vitro* HDAC inhibitory activity of the compounds **1-5** were also evaluated on these two cell lines in comparison with vorinostat as the reference drug (Table 1). Alopecurone J (4) was the most potent HDAC inhibitor with IC_50_ values of 0.08 and 3.85 μM against HCT116 and HeLa cells, respectively which were comparable to that of vorinostat (IC_50_ = 0.05 in HCT116 and 0.06 in HeLa). Since, these results could be affected by peptidase inhibitory activity of the test compounds and because there are already numerous natural products, known to inhibit trypsin we analyzed the compounds by an *in-vitro* trypsin inhibition assay (21, 22). According to this assay, none of the compounds show a trypsin inhibitory or fluorescence-based assay that reflecting real HDAC inhibitory activity of compounds. Furthermore, Lipinski’s rule of 5 is a rule in drug discovery that evaluate drug-likeness properties of chemical compounds. These molecular properties are important for drug’s pharmacokinetics including absorption, distribution, metabolism and excretion (ADEM). Based on Lipinski’s rule, a drug molecule should have a molecular mass less than 500 daltons, maximum 5 Hydrogen bond donors, maximum 10 hydrogen bond acceptor, and log P (octanol/water partition coefficient) not greater than 5. If a compound has more than two violations of these criteria, probably it would not be orally active (23, 24). The number of the rotatable bonds is a simple parameter that measures molecular flexibility. This parameter is a good descriptor of oral bioavailability (25). Molecular polar surface area (PSA) is a good factor that characterized drug absorption and blood-brain barrier penetration (26). The results of our analysis showed that the tested compounds passed four criteria of Lipinski rule. The molecular docking is the most frequently computational method in structure-based drug discovery, due to its ability to predict the mode of interaction of ligands to active sites of proteins. Drug-like properties and docking scores of compounds **1-5** with HDAC8 are shown in Table 2 Among these, compound **4** possess similar negative binding energy (8.7 kcal/mol) to positive control (vorinostat−8.6 kcal/mol).

## Discussion

Based on these biological results, it can be concluded that prenylated flavonoids may play a pivotal role in the cytotoxic activity of *S .pachycarpa.* Shirataki and coworkers have previously reported that isoprenoid substituted flavonoids induced apoptotic cell death and introduction of lavandulyl group at A ring enhanced cytotoxicity significantly (27). In alopecurone J, resveratrol is condensed to the A-ring of the flavanone (Figure 1), A-ring additionally bears an 8-C-lavandulyl substituent, and the B-ring is 2’, 4’-dioxygenated. In a study by Kang and colleagues, results demonstrated that the lavandulyl side-chain was essential for the activity of the flavonoids isolated from *S. flavescens,* which may be used as cancer chemotherapeutic and chemopreventive agents (28). In 2004, a research group investigated 11 isoflavonoids from *Sophora* species for their cytotoxic activity against two human tumor cell lines and the results showed that compounds with 2 isoprenyl groups (one in A-ring and the other in B-ring), such as isosophoranone possessed relatively higher cytotoxic activity (27, 29). The results of HDAC inhibition assay suggested that bulky hydrophobic group (1avandulyl) on A-ring and hydroxyls or a 2, 4-dihydroxybenzoyl moiety as hydrophilic group on B-ring might markedly enhance their binding affinity with cell membrane. 

**Figure 2:**
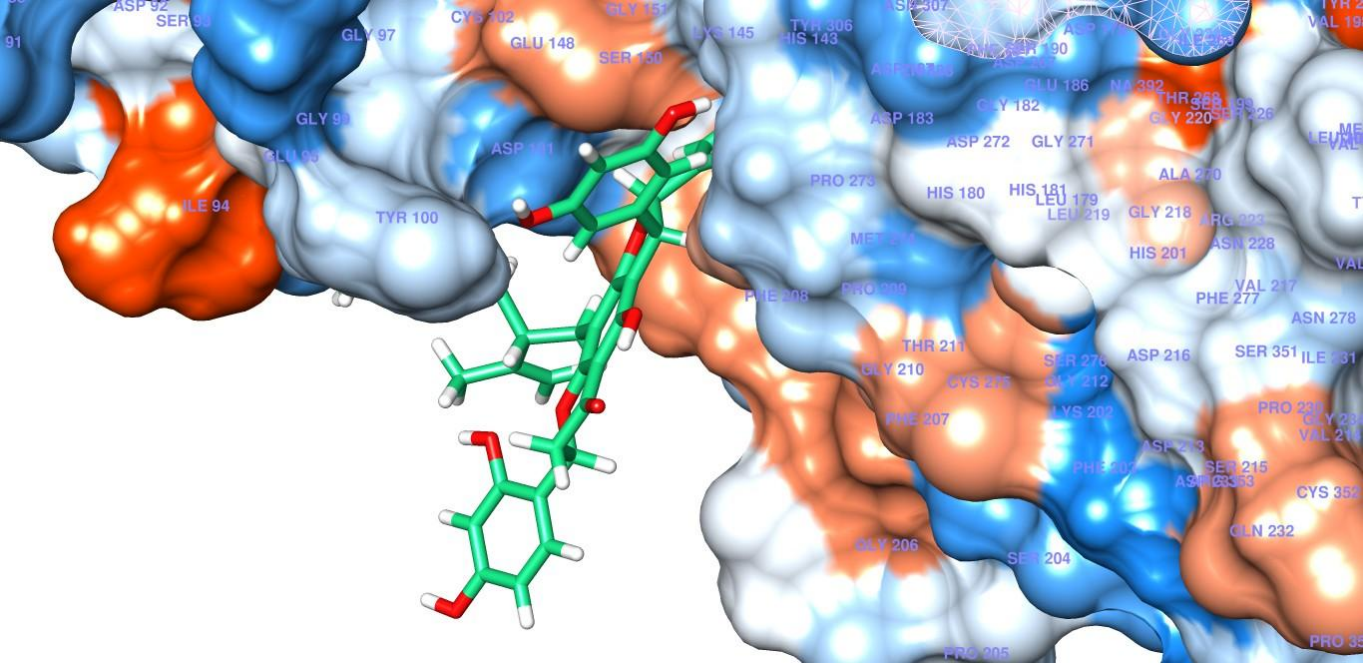
Docking model structure of compound **4** into the active site of HDAC8 protein binding pocket

**Figure 3:**
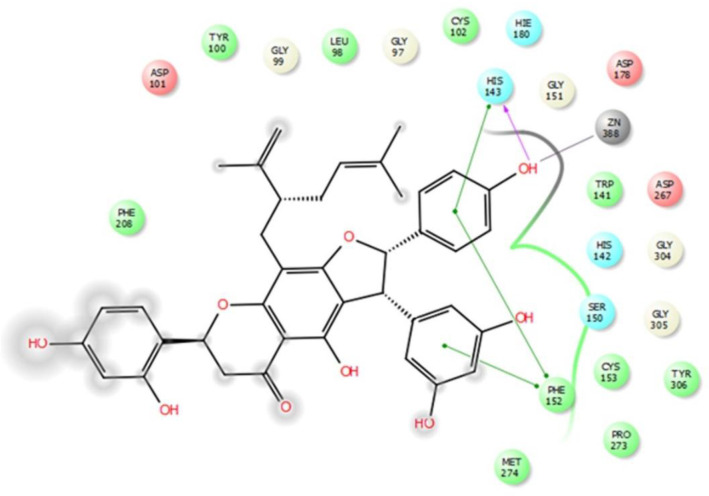
Binding model of compound **4** into active pocket of HDAC8

These computational results complied with the experimental values, and persuaded investigation of interactions between compound **4** and HDAC8 in detail. According to the experimental test alopecorune J (4) was the most potent HDAC inhibitor among the tested compounds as well as vorinostat. Molecular docking study shows that resveratrol group in compound **4** takes hydrophobic interactions with HDAC8 amino acids, such as Phe 152, His 143. Furthermore, hydroxyl moiety of this residue has metal coordination with ZN that plays vital roles in protein inhibition (Figures 2, 3). 

These results are in agreement with those observed from the native ligand trichostatin A (co-crystallized ligand in RCSB Protein Data Bank) with common residues of Phe152, Asp101, Trp141, His142, Met247. In conclusion, alopecurone J (4) was the best HDAC inhibitor among the isolated compounds. Molecular modeling study demonstrated that the resveratrol group played a vital role in HDAC inhibition through the hydrogen bond and metal coordination to zinc ion. 

**Table 1:** **Cytotoxic and HDAC inhibition IC50s (μM) of compounds **1-5** and vorinostat against human colon cancer (HCT116), human cervical (HeLa), lung (A549) and ovarian cancer (A2780) cell lines**

**Compound**	**Cytotoxicity IC**_50_				**HDAC inhibition**	
	HeLa	HCT116	A2780	A549	Hela	HCT116
1	43.16	39.27	49.27	53.45	34.7	26.46
2	44.22	54.44	65.53	72.50	>70	>70
3	69.42	53.96	>100	76.54	>70	>70
4	9.97	17.96	21.90	30.91	3.85	0.08
5	52.57	48.44	69.01	54.89	24.9	22.18
Vorinostat	2.43	2.98	8.02	6.83	0.06	0.05

**Table 2:** **Drug likeness properties of the 1-5 compound and docking energy**

compound	cLogP	H-donor	H-acceptor	PSA	MW	Number of rotatable bonds	Docking energy
1	4.772	2	4.75	88.406	438.519	5	-5.600
2	3.959	3	4.75	106.285	424.493	6	-6.900
3	0.737	5	4.7	91.771	260.246	1	-7.463
4	5.169	5	7	156.443	650.724	8	-8.743
5	4.24	4	7	163.022	582.606	5	-7.346
**vorinostat**	0.415	3	6.7	103.595	264.324	10	-8.646

## Conclusion

In the present study alopecurone J was the most active with IC_50_ values in the range of 9.97−30.91 μM against four cancer cell lines with potent pan-HDAC inhibitory activity (IC_50_ = 0.08−3.85 μM). Molecular docking experiments of these compounds with HDAC8 displayed potential selective HDAC inhibitory. Molecular docking data showed consistent results in the *in-vitro *experiments with high selectivity towards HDAC8. The resveratrol group plays an important role in HDAC inhibition.

## Supplementary material

Supplementary material related to this article is available online, alongside Figures S1–S6.

## Funding

This research was financially supported by grants from the Mashhad University of Medical Sciences Research Council [grant number 958763].
